# Changes in dynamics of tumor/endothelial cell adhesive interactions depending on endothelial cell growth state and elastic properties

**DOI:** 10.1371/journal.pone.0269552

**Published:** 2022-06-06

**Authors:** Leike Xie, Zhe Sun, Nicola J. Brown, Olga V. Glinskii, Gerald A. Meininger, Vladislav V. Glinsky

**Affiliations:** 1 Dalton Cardiovascular Research Center, University of Missouri, Columbia, Missouri, United States of America; 2 Department of Pathology and Anatomical Sciences, University of Missouri, Columbia, Missouri, United States of America; 3 Microcirculation Research Group, Department of Oncology and Metabolism, Faculty of Medicine, Dentistry and Health, University of Sheffield, Sheffield, United Kingdom; 4 Department of Medical Pharmacology and Physiology, University of Missouri, Columbia, Missouri, United States of America; 5 Research Service, Harry S. Truman Memorial Veterans Hospital, Columbia, Missouri, United States of America; Eötvös Loránd Research Network Biological Research Centre, HUNGARY

## Abstract

Cancer cell adhesion to the endothelium is a crucial process in hematogenous metastasis, but how the integrity of the endothelial barrier and endothelial cell (EC) mechanical properties influence the adhesion between metastatic cancer cells and the endothelium remain unclear. In the present study, we have measured the adhesion between single cancer cells and two types of ECs at various growth states and their mechanical properties (elasticity) using atomic force microscopy single cell force spectroscopy. We demonstrated that the EC stiffness increased and adhesion with cancer cells decreased, as ECs grew from a single cell to a confluent state and developed cell-cell contacts, but this was reversed when confluent cells returned to a single state in a scratch assay. Our results suggest that the integrity of the endothelial barrier is an important factor in reducing the ability of the metastatic tumor cells to adhere to the vascular endothelium, extravasate and lodge in the vasculature of a distant organ where secondary metastatic tumors would develop.

## Introduction

Vascular endothelial cells (ECs) that physiologically form a monolayer lining the interior of blood vessels serve as a dynamic barrier controlling cell and molecule movement into and out of the blood stream [1, 2]. Due to the location and function, the endothelial barrier plays an important role in hematogenous cancer metastasis [3]. Hematogenous cancer metastasis is a complex process involving several major steps, when tumor cells directly interact with ECs including blood borne metastatic tumor cell arrest in a distant organ vasculature and tumor cell extravasation out of the blood stream into the surrounding tissue where metastases may develop [4–7].

Currently, there is a certain degree of controversy regarding how the integrity of the endothelial barrier regulates metastasis. Several studies have reported that the endothelium functions as a potential barrier against tumor cell invasion and the latter is facilitated when the integrity of the endothelial barrier is disrupted, but prevented when it is enhanced [8–10]. In contrast, other studies have shown that the endothelium can induce tumor cell invasion [11, 12]. Thus, interactions between tumor and endothelial cells are complex, multifaceted and dependent on many different factors influencing both tumor cells and ECs including EC mechanical properties. Changes in EC mechanical properties have a significant impact on important endothelial functions [13–15]. With regards to tumor-endothelial cell interactions, it has been demonstrated that invasive breast tumor cells co-cultured with endothelium regulate EC mechanical properties by reducing their stiffness and increasing cytoskeletal remodeling dynamics [16]. These findings implicate cancer cells in the disruption of the endothelial barrier by way of altering the endothelial stiffness, and suggest the importance of EC mechanical properties in the trans-endothelial migration of cancer cells during extravasation [17]. However, whether and how EC mechanical properties effect initial adhesive interactions between tumor cells and ECs is unknown. Previously, studies have been carried out by several groups to determine adhesion dynamics between tumor cells and ECs [18–23] as well as the biomechanical properties of ECs isolated from distinct tissues [24–29] using various techniques including atomic force microscopy (AFM). However, there are very few studies characterizing EC adhesive and mechanical properties at different states of growth. We hypothesize that the stiffness of ECs will increase with cell confluency, while adhesive forces between ECs and cancer cells will exhibit reciprocal dynamics.

To test our hypothesis, in this study we cultured two types of ECs, immortalized human bone marrow endothelial cells and primary human pulmonary microvascular endothelial cells, in four growth states (non-confluent, sub-confluent, confluent and migrating) that partially imitate the varied integrity status of the endothelial lining in blood vessels. We examined the effect of cell confluency on the measured EC stiffness using AFM, an indispensable tool for investigating the mechanical properties, surface topography and adhesive interactions of living cells [19, 30–32]. The adhesion dynamics of ECs interacting with human breast cancer cells for three different cell contact times were also evaluated using the AFM single-cell force spectroscopy method, where a single cancer cell is attached to the tip of a cantilever and brought into contact with EC.

## Materials and methods

### Cell culture and preparations

The human bone marrow endothelial cell line HBMEC-60, kindly provided by Dr. C. E. van der Schoot (University of Amsterdam, Amsterdam, The Netherlands), has been described previously [33] and was maintained in Medium 200 (Invitrogen, Carlsbad, CA) supplemented with 20% fetal bovine serum (FBS, Atlanta Biologicals, Lawrenceville, GA, USA) and low-serum growth supplement (Invitrogen) in collagen-I coated Petri dishes (BD Bioscience) [23, 34]. Primary human pulmonary microvascular endothelial cells (HPMEC) were purchased from ScienCell Research Laboratories (Carlsbad, CA) and cultured in Endothelial Cell Medium (ECM, ScienCell Research Laboratories) containing 5% FBS and endothelial cell growth supplement (ECGS) in fibronectin coated dishes as recommended by the company. HPMEC at passages 3–5 were used for experiments. Metastatic human triple negative breast cancer cell line MDA-MB-231 (MB231) was purchased from the American Type Culture Collection (ATCC, Manassas, VA) and routinely grown in RPMI 1640 medium (Invitrogen) supplemented with 10% FBS and L-glutamine. All cells were maintained as monolayer cultures in a humidified incubator (Heraeus Instruments, Newtown, CT) in 5% CO_2_ at 37°C.

**Fig 1:**
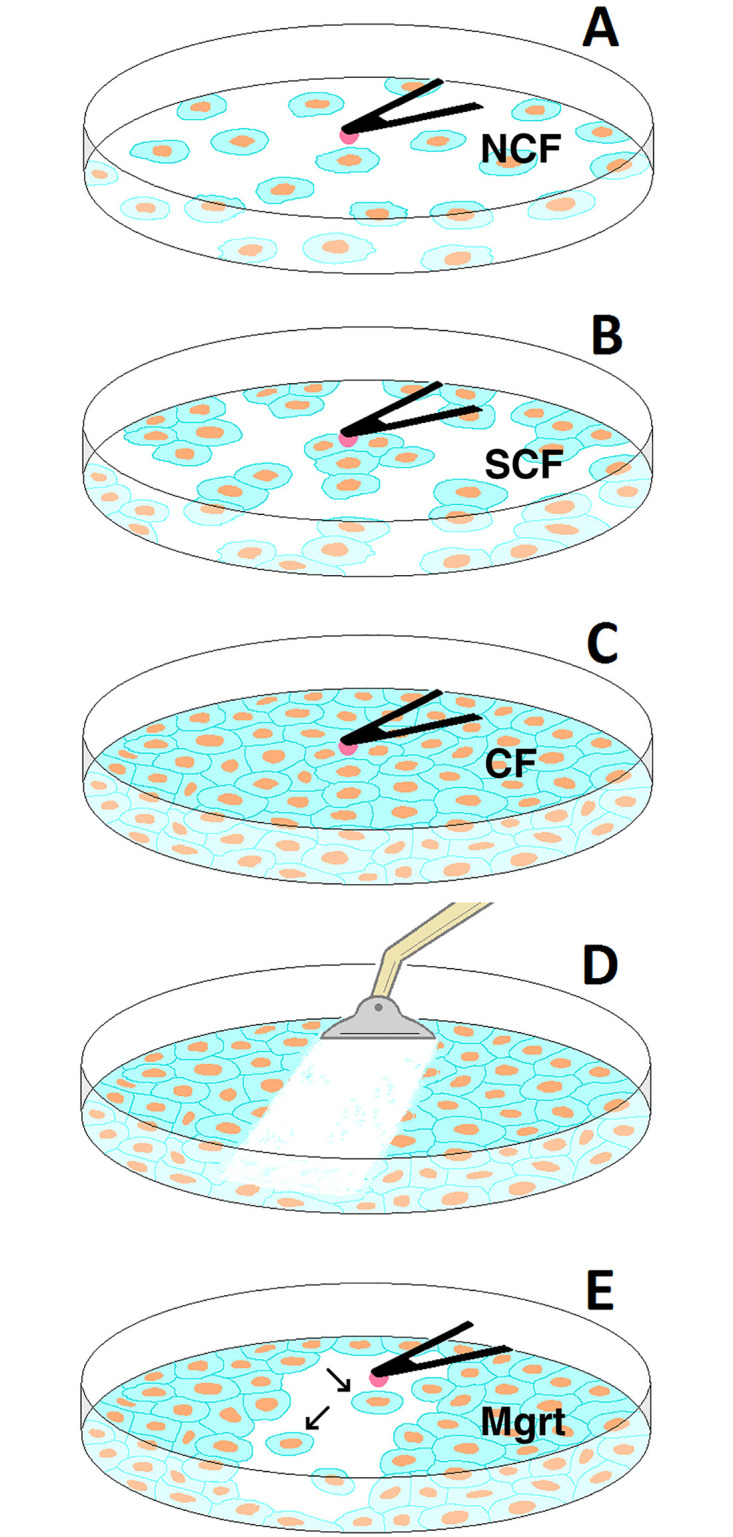
Diagram of EC growing states and AFM probing. Endothelial cells (green and orange) were cultured at NCF (A), SCF (B); and CF (C) status. For acquisition of Mgrt cells, a scratch in a straight line on EC monolayer was created using a cell scraper (D). After continued culture for two more days, individual Mgrt cells (indicated by arrows in E) from the leading edge of the scratch appeared in the scratched area. Cells at different status were probed by either tipped cantilever for EC stiffness measurement and topography, or by tipless cantilever (black triangle in A-C & E) coupled with aMB231 cell (pink in A-C & E) for measuring cancer cell-EC adhesion.

HBMEC-60 and HPMEC cells were plated in collagen-I coated 60 mm dishes, and grown for two to three days to achieve the desired growth status (Fig 1A–1C), i.e. non-confluence (NCF, Fig 1A), sub-confluence (SCF, Fig 1B) and confluence (CF, Fig 1C) or full-confluence. To investigate migrating (Mgrt, Fig 1E) cells, a scratch assay model was used [35]. A sterile cell scraper (BD Falcon, Tewksbury, MA) with a trimmed ~0.6 mm wide blade was used to scratch confluent endothelial monolayers in a straight line (Fig 1D), followed by a wash with growth media. After continuing culture for a further two days, individual cells that migrated from the leading edge of the scratch appeared in the gap area (Fig 1E). In order to avoid possible variance introduced by cell passage and culture period, cells at a similar range of passages were used and seeded at different initial densities to produce the desired degree of confluence within the same time period in culture (2–3 days). At the time of the AFM experiment, cells covering 20–30% of the area of a petri dish were used for NCF to enhance the probability of obtaining single cells in culture without any intercellular contact with neighboring cells. Cells with coverage at 50–70% with clusters formed by four to five adjacent cells in contact with each other were used for SCF. In SCF clusters, only the cells in contact with neighbors but still having one-third to one-half of free margin were selected as SCF cells. Cells forming a monolayer with nearly 100% of dish coverage were considered as CF. CF cells reached a monolayer (without piling) within 16 hours prior to the experiment. Before each experiment, cell growth media was replaced with serum-free, CO_2_ independent medium (CO_2_-IM, Invitrogen).

MB231 cells at 40–50% confluence were detached in Cell Dissociation Buffer (Invitrogen) before each experiment. After centrifugation, cells were re-suspended in CO_2_-IM and used for AFM cantilever attachment. All cancer and endothelial cells were allowed to equilibrate for fifteen minutes in CO_2_-IM in the atmosphere at room temperature prior to AFM experiments, and continuously maintained at the same conditions during the experiments. Following AFM experiments, the EC viability was >95%.

All AFM experiments, including monitoring of mechanical properties, topological scans of cells, and measurements of cell-cell adhesion were performed using an Asylum Research AFM System (Model MFP-3D-BIO, Asylum Research, Santa Barbara, CA) with IGOR Pro software (WaveMetrics Inc., Oregon). The system was mounted on an inverted optical microscope (Model IX81, Olympus America Inc.).

### EC stiffness characterization and topography imaging

**Fig 2:**
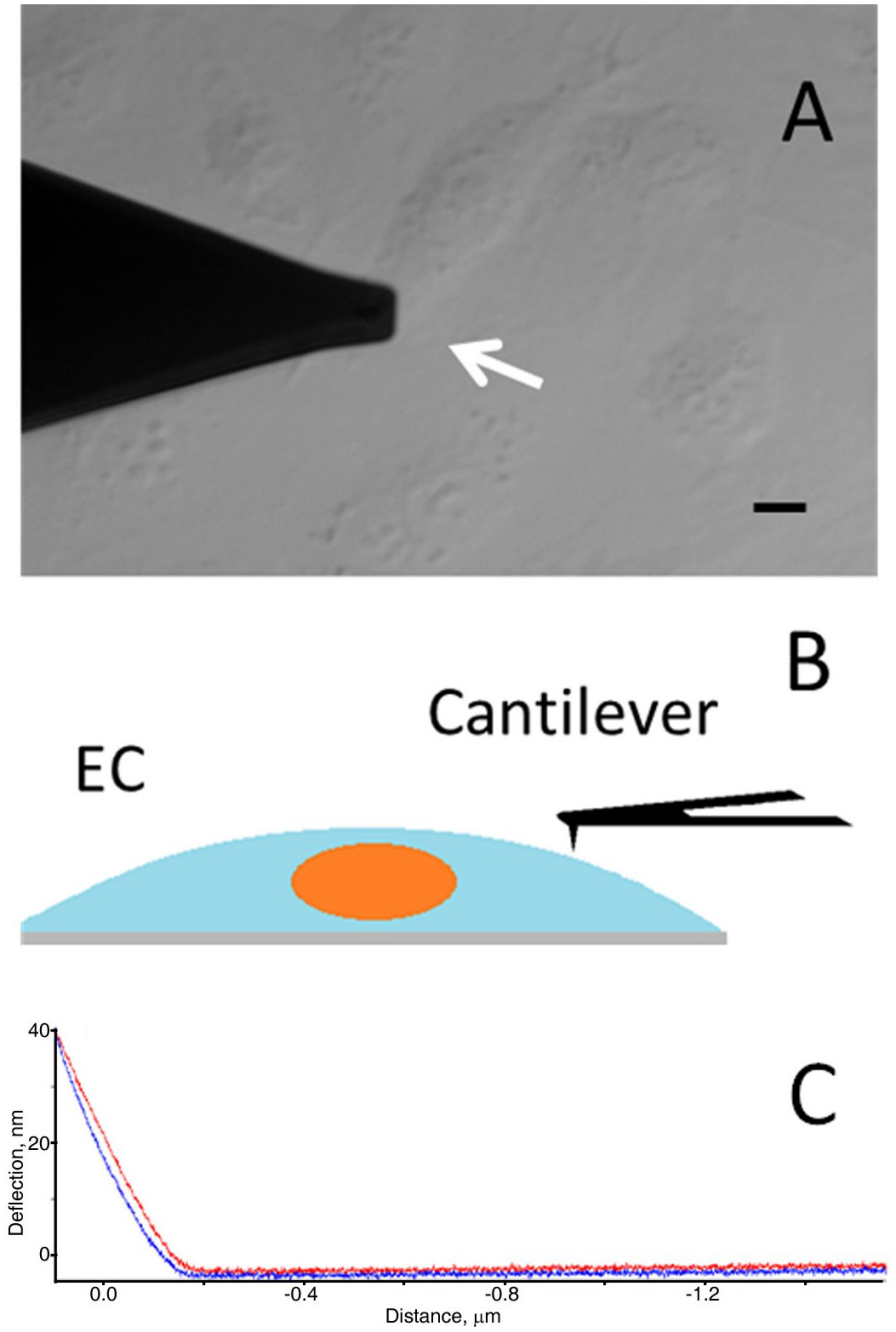
Representative pictures (A, C) and diagram (B) of the approach for quantitating cell stiffness. EC stiffness was measured by indenting a HBME cell on the cell cortex (arrow in A) with the tip at the end of the cantilever (A and B). The corresponding force curve included approach (red in C) and retraction (blue in C) curves that show cantilever deflection. Cell stiffness (Young’s modulus) was converted from the approach curve. Scale bar in A = 10 μm.

A stylus-type silicon nitride cantilever (MLCT, Bruker Corp., Santa Barbara, CA.) with a tip radius of 20–60 nm, tip height of 2.5–8.0 μm and nominal spring constant of 0.01 N/m was used to measure the cell stiffness and scan cell images in ECs. Cantilevers were calibrated after a given experiment using the thermal noise method [36]. A Petri dish containing ECs in CO_2_-IM was placed under the AFM. Cells were randomly selected and indented at a site midway between the nucleus and cell margin, with an average depth of 204.63±74.87 nm. For elasticity measurements, ECs were chosen randomly at least 20 cells and /or 400 μm apart. The midway between the nucleus and cell margin was chosen as the testing site to ensure the consistency of the experiments, as the area above the nucleus is least variable in terms of elasticity while the status of cortical cytoskeleton and stiffness on the very periphery of the cells could be effected by EC interactions with neighboring cells in confluent and sub-confluent cultures as compared with single cell states (NCF and Mgrt cells). EC stiffness was determined by a continuous nanoindentation protocol at 0.3 Hz sampling frequency, with an approach/retraction velocity of 0.3 μm/sec and a loading force of 500 pN (Fig 2). In each group, at least fifty cells were randomly selected for elasticity measurement and cell topography scanning. Ten to fifteen force curves were collected on each cell. The curves were analyzed using MATLAB software (version R2010b, MathWorks Inc., Natick, MA, USA). Young’s modulus (kPa) representing EC elastic stiffness was calculated from the approach force curves based on a modified Hertz model [37]. The mean of stiffness values for single cells was calculated and then averaged together for each group. Live cell topography imaging was performed in contact mode. The digital density of the scanned area was 256 x 256 pixels. Both deflection and height image data were collected.

### Cantilever functionalization and cell attachment

A tipless nitride cantilever (MLCT-O10, Bruker Corp.) with a nominal spring constant of 0.01 N/m was used for measurements of cell-cell adhesion force. Cantilever calibration was carried out using the aforementioned method prior to each experiment. The cantilever functionalization with biotinylated concanavalin A (Con A, Sigma-Aldrich) was performed following procedures described previously [23]. Briefly, the cantilevers were treated in 0.5 mg/ml biotin labeled BSA (Sigma-Aldrich) overnight, followed by incubation in 0.5 mg/ml streptavidin (Sigma-Aldrich) for 20 min and then in 0.5 mg/ml Con A for 20 min at room temperature and rinsed in PBS. The functionalized probe was mounted on the AFM head and placed into a culture dish which contained EC in CO_2_-IM on the microscope stage. MB231 cells were injected into the dish. Cancer cell to cantilever attachment was performed manually and controlled by cantilever tip engagement in the AFM software to maintain the given force to the cell at ≤ 500 pN. Single cancer cells of similar sizes (12–12.5 μm in diameter) were selected. The tip of the Con A-coated cantilever was aligned with the center of the selected cell and gently lowered onto the cell for 1 sec. The cancer cell was lifted by cantilever retraction and allowed to rest for 5 to 10 minutes to establish firm cell-cantilever attachment.

### Cell-cell adhesion measurement

**Fig 3:**
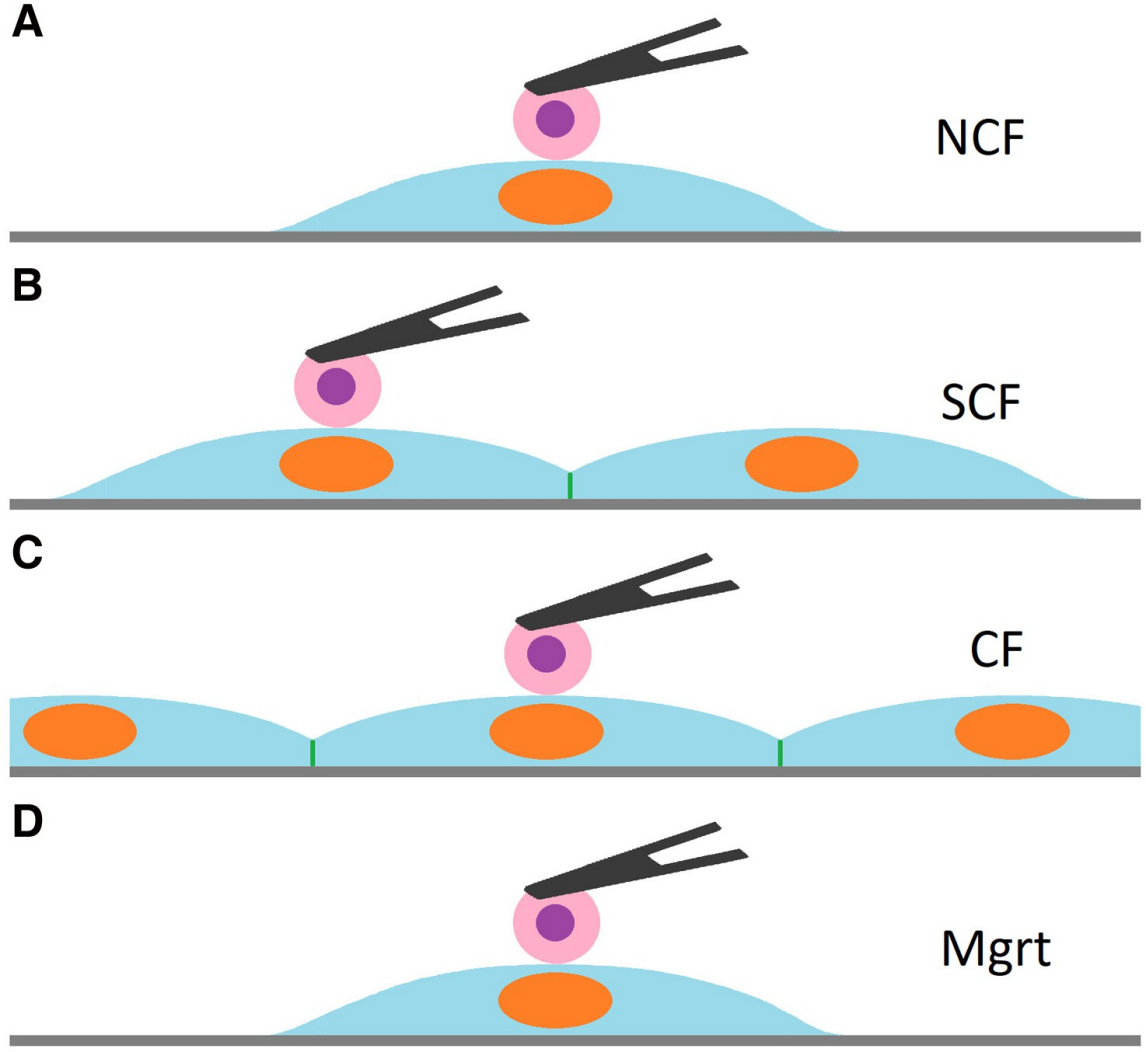
Detection of cancer cell adhesion to ECs at four different growth states. To compare adhesions at different EC growth states, a MB231 cell (Pink with purple) attached to the cantilever (black triangle) was brought into contact with non-confluent (NCF) EC (A), sub-confluent (SCF) EC (B), confluent (CF) EC (C) and migrating (Mgrt) EC (D) over the EC nucleus for three different cell contact times (0.5, 10 and 60 sec).

To measure the adhesion between MB231 cells and ECs, the single tumor cell attached to the tip of the cantilever was positioned over the specific site on the EC and then brought into the contact with the endothelial cell for a defined time period. To ensure the consistency between the experiments with ECs at different growth states, all adhesion measurements were performed over the EC’s nuclear area (Fig 3). For adhesion measurement, at least four independent experiments were performed for each experimental setting with at least twelve ECs tested for interactions with tumor cells in each experiment. The speed of the cantilever movement was set at 1.60 μm/sec, with a cell-cell contact force of 500 pN. The maximum range of cantilever force distance approach-retraction was near 40 μm, which separated the tumor cell from the ECs as completely as possible when the cantilever retracted. The closed-loop feedback mode was enabled by default to control the position of the measurement [23]. Forces were determined by cantilever approach-retraction cycles, through which the MB231 was brought into contact with the EC nuclear area. Three contact periods (0.5, 10 and 60 sec) were set and ten curves were collected for each contact time point.

Adhesion forces were calculated in pN from retraction curves in MATLAB software. The overall pulling force that was required to completely separate the cancer cell from EC was defined as the total adhesion force, which included rupture adhesion forces that represented individual forces to break single ligand-receptor bonds [23]. The mean total adhesion forces for single cancer cells were calculated and further averaged together for each group. The distributions of rupture forces were plotted in Microsoft Excel.

### Cortical actin fluorescent staining and imaging

HBMEC-60 and HPMEC cells were cultured in chamber slides (Lab-Tek II, Cat# 154526) coated with collagen-I (BD Biosciences, Cat# 354236) as described in Cell culture and preparations section. When desired growth states (NCF, SCF, CF, Mgrt) were reached, cells were fixed in 2% paraformaldehyde (PFA) by adding equal amount of 4% PFA to the medium. Following rinse in phosphate buffered saline (PBS, pH 7.4), cells were permeabilized in 0.1% Triton X-100 for 5 minutes, then incubated with Alexa Fluor 647 conjugated Phalloidin (Cell Signaling, 8940) at a dilution of 1:200 in PBS for 30 minutes to label actin filaments (pseudo green). After additional rinse with PBS, samples were mounted with antifade mounting medium containing Propidium Iodide (Vector Laboratories, H-1300) to counterstain cell nuclei (red). Images were acquired at 60x magnification on confocal FluoView FV1000 inverted microscope system (Olympus) equipped with FV10-ASW software. The Z-stacks (42–62 planes per stack) were acquired with 0.2 μm step size. ImageJ2, version 2.3.0/1.53f software was used to generate Max Intensity image from each stack.

### Statistical analysis

Data are expressed as a mean ± standard error of the mean (SEM). Cell stiffness data for four growth states, in addition to cancer cell-EC adhesion data for different growth states were compared using *t*-test and ANOVA analysis. A value of P< 0.05 was considered significant for the comparisons. The regression analysis of the relationship between tumor cell-EC adhesion and EC elasticity was performed in Microsoft Excel.

## Results

### Endothelial cell elasticity changes with EC confluency (growth state)

**Fig 4:**
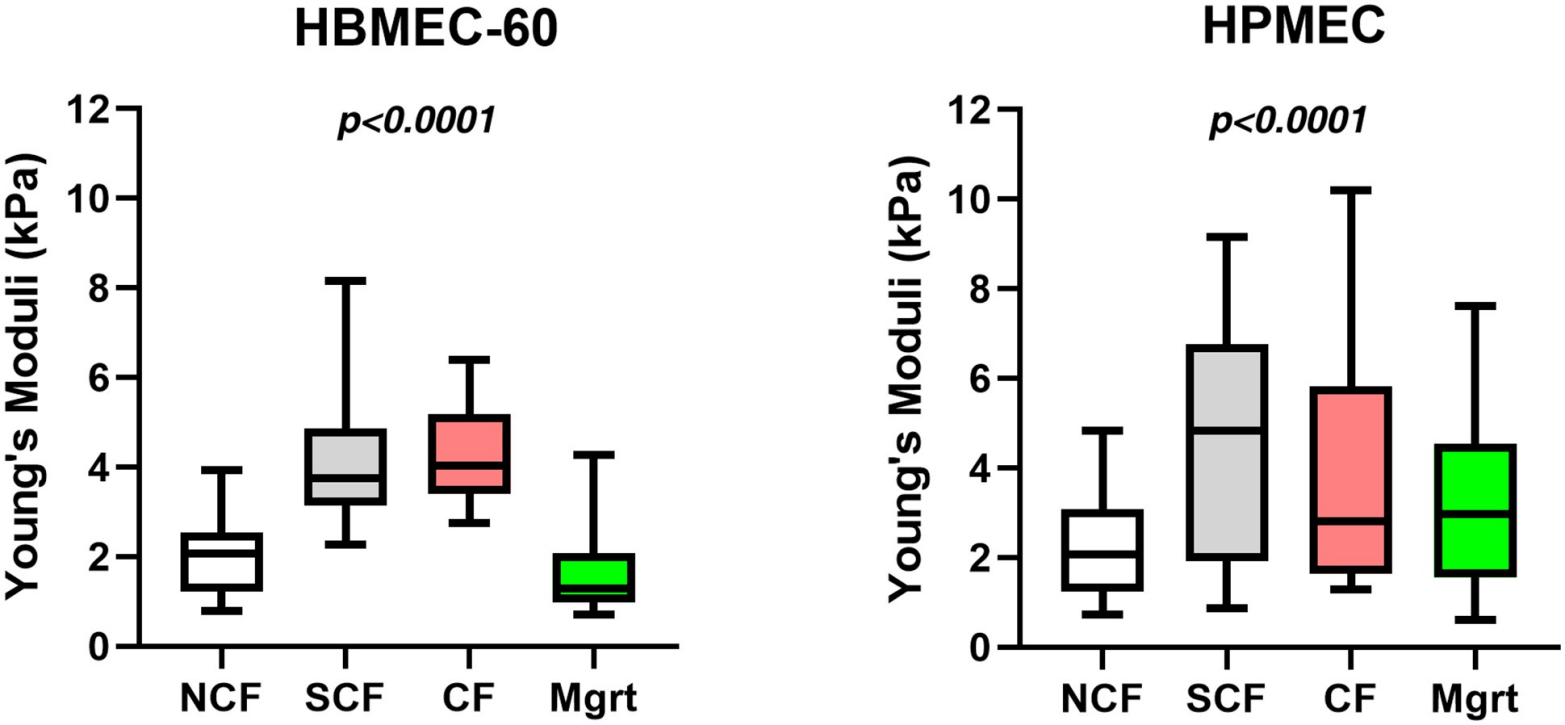
EC cortical stiffness at four different growth states. Box diagrams showing changes in cortical stiffness (Young’s moduli) of HBMEC-60 (left panel) and HPMEC (right panel) endothelial cells at four different growth/confluency states, NCF (non-confluent), SCF (sub-confluent), CF (confluent) and Mgrt (migrating). Whiskers, 5%– 95%. *P*–Ordinary One-Way ANOVA.

The cortical stiffness of ECs was measured and compared for different degrees of confluence (growth state) using the AFM indentation approach (Fig 4). In HBMEC-60 cells, Young’s elastic modulus was 2.03 ± 0.21 kPa (n = 50) in NCF state, whereas in SCF (4.29 ± 0.33 kPa, n = 54) and CF (4.25 ± 0.57 kPa, n = 66) the elastic modulus was significantly increased by more than two-fold compared to the NCF (p <0.01 for both). The SCF and CF cells had similar values of Young’s moduli, with no significant difference between the two. Mgrt cells (1.75 ± 0.25 kPa) in scratch assay showed a slightly lower stiffness than the NCF cells, but there was no statistical difference between the two single cell states in culture.

In the HPMEC, Young’s moduli of both SCF (4.75 ± 0.43 kPa, n = 70) and CF (3.91 ± 0.42 kPa, n = 99) cells were also significantly (p< 0.001 for both) higher than that of the NCF cells (2.33 ± 0.31 kPa, n = 79). There was no significant difference between the SCF and CF cells. Mgrt HPMEC stiffness (3.30 ± 0.28 kPa, n = 81) was higher (p< 0.01) than in NCF HPMEC and lower (p< 0.01) than in SCF cells. Collectively, these results demonstrate that the cortical stiffness of ECs increases when cells progress from a single cell state to SCF state, but does not increase further with EC confluency changing from the SCF to the CF state.

### EC cytoskeleton reorganization visualized by AFM topography imaging and confocal microscopy

**Fig 5:**
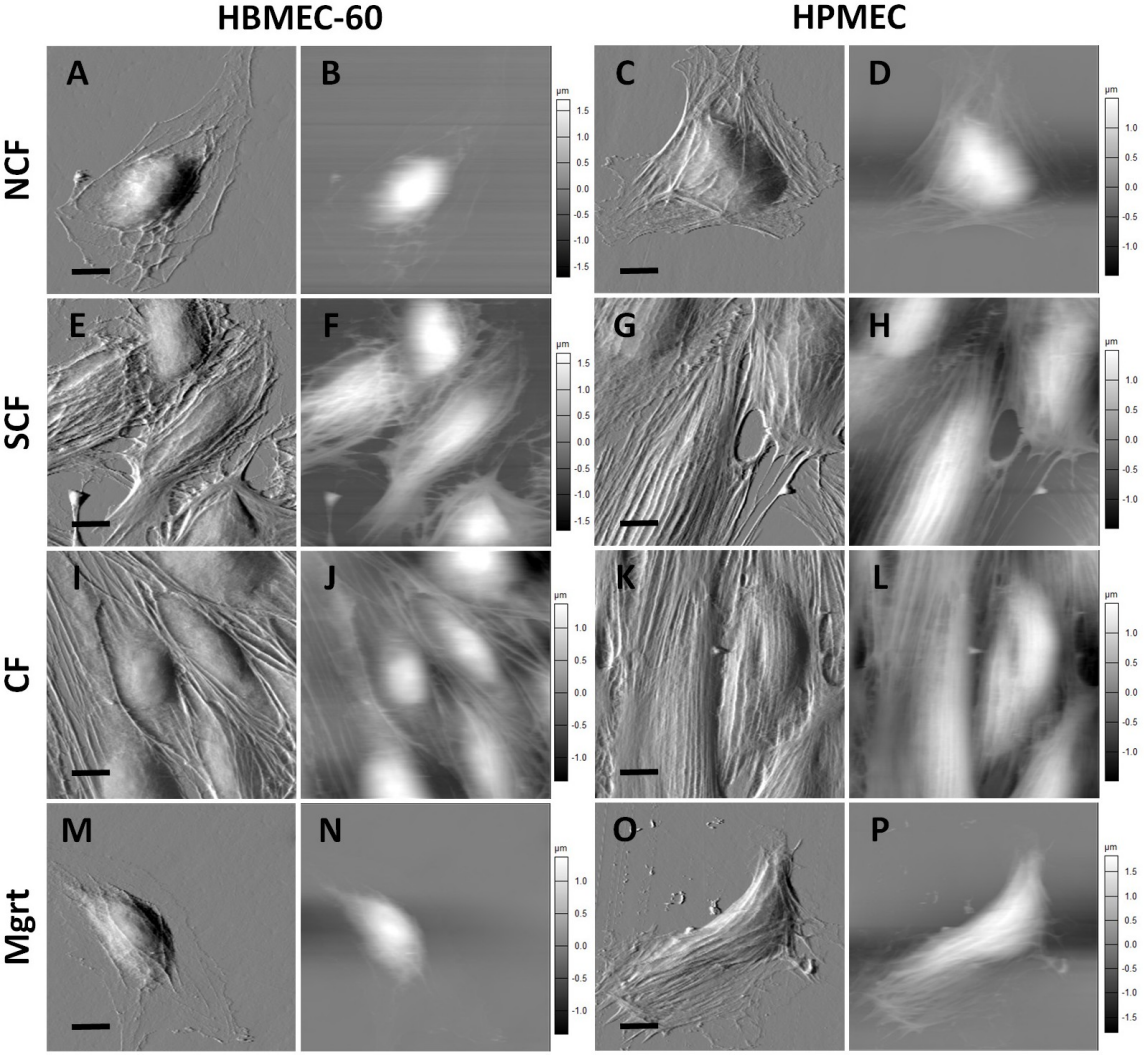
Representative topography imaging (60 μm x 60 μm) of ECs at different growing states. Cantilever deflection images (A, E, I and M for HBMEC-60; C, G, K and O for HPMEC) and cell height images (B, F, J and N for HBMEC-60; D, H, L and P for HPMEC) on ECs were performed to visualize the EC cytoskeletal structure underneath cell membrane. Both HBMEC-60 (E and I) and HPMEC (G and K) cells at SCF state revealed denser, thicker and more aligned stress fiber networks, compared to their respective NCF (A and C) states. The Mgrt (M) cell in HBMEC-60 showed less stress fibers than the NCF (A) cells, while in HPMEC, the Mgrt (O) cells retained more fibers inside the cell. There were no remarkable changes seen in cell height as a function of confluency in either HBMEC-60 (B, F, J and N) or HPMEC (D, H, L and P). Gray vertical scale bars (μm) are for cell height. Black scale bars = 10 μm.

**Fig 6:**
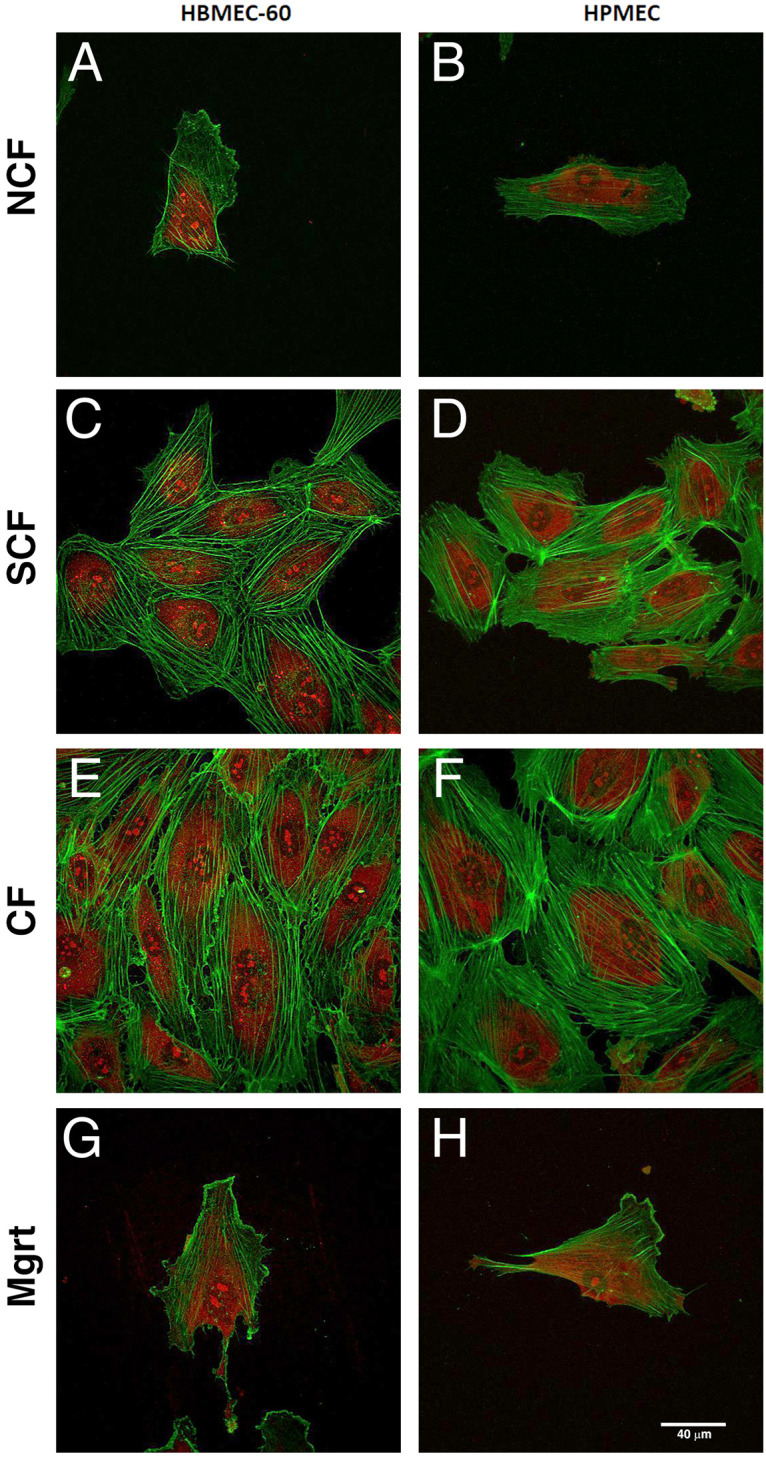
Representative images of EC cortical actin cytoskeleton at different growing states visualized by Phalloidin staining and confocal microscopy. HBMEC-60 (A, C, E and G) and HPMEC (B, D, F and H) cells were cultured in chamber slides until reaching desired growth states (NCF, SCF, CF, Mgrt). Cells were fixed in 2% paraformaldehyde and permeabilized with 0.1% Triton X-100 as described in Materials and Methods. Cellular actin was visualized using Alexa Fluor 647 conjugated Phalloidin (pseudo green) and nuclei counterstained with Propidium Iodide (red). The Z-stacks (42–62 planes per stack) were acquired with 0.2 μm step size at 60x magnification and Max Intensity images were generated from each stack. Both HBMEC-60 and HPMEC cells at SCF (C and D) and CF (E and F) states revealed denser, thicker and more aligned stress fiber networks, compared to their respective NCF (A and B) and Mgrt (G and H) states. Scale bar in H = 40 mm.

Cell elasticity is highly correlated with the state of the intracellular cytoskeleton and cortical actin stress fibers which can be visualized using AFM topography [13, 31]. In our experiments, EC cells in all four states were analyzed using high resolution AFM topography imaging in contact mode to observe the cell cortical cytoskeletal structure underneath the cell membrane. Fig 5 shows both cantilever deflection (A, E, I and M for HBMEC-60; C, G, K and O for HPMEC) and cell height (B, F, J and N for HBMEC-60; D, H, L and P for HPMEC) images with a scanning size of 60 × 60 μm. The HBMEC-60 cells in the NCF state were characterized by a thin cytosolic content with narrow cytoskeleton networks underneath the membrane (Fig 5A). A few stress fibers were observed stretched from the central area of the cell to the peripheral cell protrusions. In contrast, at the SCF and CF states the cell stress fibers demonstrated increased density and thickness, were more elongated and aligned, with a proportion covering the cell nucleus and obscuring the protrusion (Fig 5E and 5I). The Mgrt cell (Fig 5M) revealed less stress fibers than the NCF cells (Fig 5A). Similar to the HBMEC-60, HPMEC cells at SCF (Fig 5G) and CF (Fig 5K) states demonstrated denser, more elongated, aligned and nucleus-covering stress fibers inside the cells, compared with the NCF cells (Fig 5C). The Mgrt HPMEC (Fig 5O) appeared to retain more stress fibers than Mgrt HBMEC-60. We did not see pronounced changes in cell height as a function of confluence in either HBMEC-60 (Fig 5B, 5F, 5J and 5N) or HPMEC (Fig 5D, 5H, 5L and 5P). These topology results indicate that EC stress fibers reorganize and remodel as the cell confluency changes. The observed changes in stress fiber density and thickness correlate with the increases in stiffness observed in SCF and CF EC states. These results were further corroborated using cortical actin cytoskeleton staining with Phalloidin followed by confocal fluorescent microscopy (Fig 6).

### MB231-EC adhesions in different EC growth states

**Fig 7:**
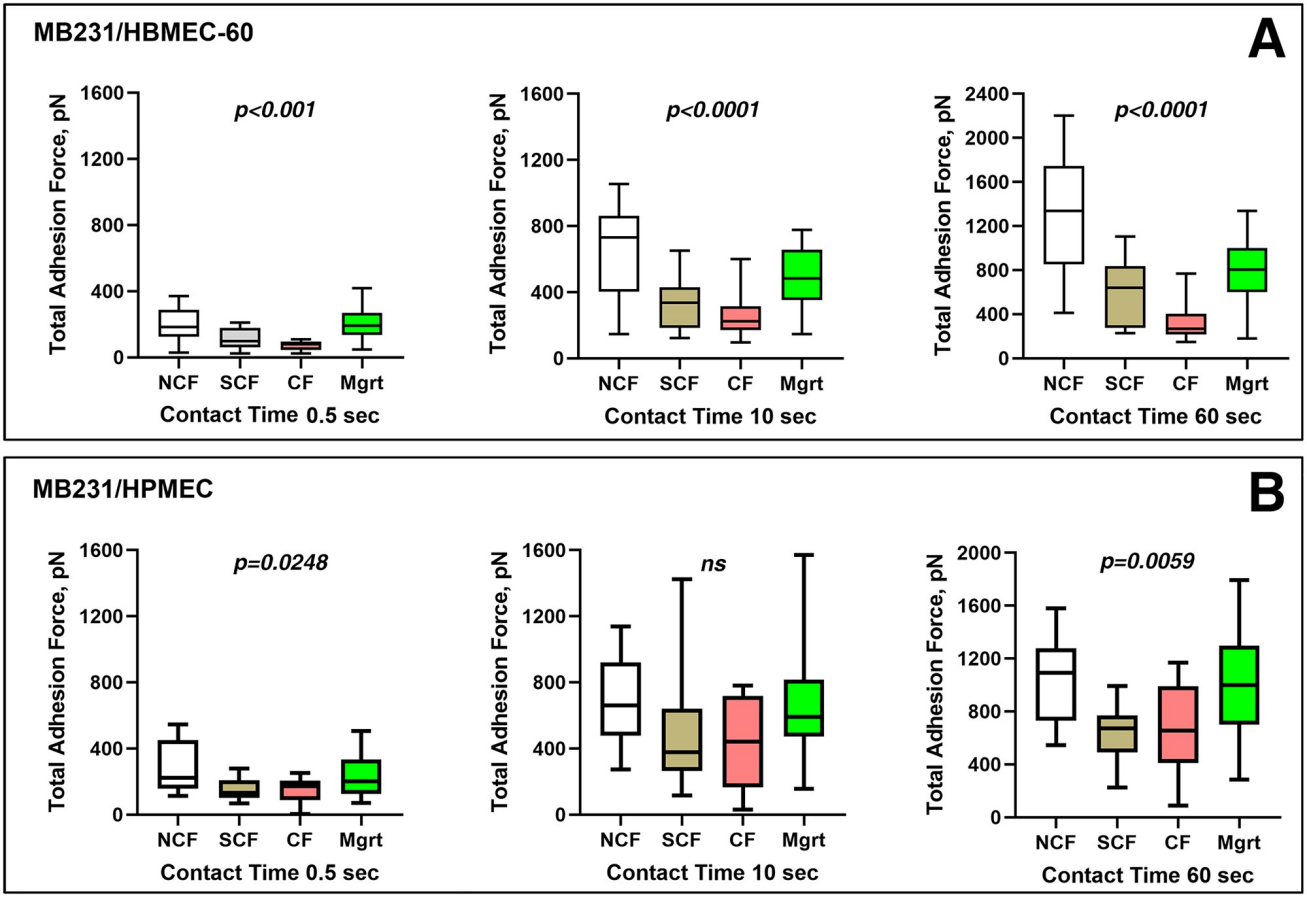
Total MB231/HBMEC-60 and MB231/HPMEC adhesion forces in different EC growing states for three cell-cell contact times. Box diagrams showing changes in total adhesion forces between MB231 human breast carcinoma cells and HBMEC-60 (top panel A) and HPMEC (bottom panel B) endothelial cells at four different growth/confluency states, NCF (non-confluent), SCF (sub-confluent), CF (confluent) and Mgrt (migrating) and three (0.5 sec, 10 sec, 60 sec) cell-cell contact times. Whiskers, 5%– 95%. *P*–Ordinary One-Way ANOVA (ns–not significant).

Using a tumor cell loaded probe to bring the cancer cell into contact with ECs via AFM cantilever approach/retraction cycles, we have analyzed real-time adhesion events between MB231 and ECs in three different EC confluence states (NCF, SCF and CF) and Mgrt state in a scratch assay for three periods (0.5, 10 and 60 sec) of cell-cell contact. The results of these experiments are plotted in Fig 7.

In MB231 to HBMEC-60 adhesion group (Fig 7A), all the highest total adhesion force values were detected with NCF HBMEC-60 cells, 200.14 ± 26.96 (n = 13), 649.69 ± 76.70 (n = 13) and 1268.08 ± 143.18 (n = 13) pN for cell-cell contact times of 0.5, 10 and 60 sec, respectively, while the lowest adhesion forces were observed with CF HBMEC-60 cells, (74.19 ± 9.98 pN, p< 0.01, n = 12), (255.03 ± 38.46 pN, p< 0.01) and (348.43 ± 51.67 pN, p< 0.01) i.e. 2.69, 2.55 and 3.64 times lower than with NCF HBMEC-60 cells for 0.5, 10 and 60 sec respectively. Total adhesion force values in the MB231 to SCF HBMEC-60 group were in between the values for NCF and CF groups at 108.10 ± 15.70 pN for 0.5 sec (n = 14), 324.65 ± 38.82 pN for 10 sec (n = 14) and 603.29 ± 75.38 pN (p< 0.05) for 60 sec (n = 14), although there were no statistical differences between SCF and CF groups for contact times of 0.5 and 10 sec. Interestingly, adhesion forces betweenMB231 cells and Mgrt HBMEC-60 cells were in a similar range as for the NCF cells for the 0.5 sec contact time (198.97 ± 29.05 pN, n = 12), but between the NCF and SCF for the 10 sec (481.44 ± 56.22 pN, n = 12), and close to the SCF for 60 sec (765.83 ± 90.19 pN, n = 12) contact times.

Similar to HBMEC-60, in HPMEC experiments (Fig 7B), the NCF cells also exhibited the greatest adhesions with MB231 cells for all three contact times: 276.11±43.56 pN for 0.5 sec (n = 12), 674.09±72.87 pN for 10 sec (n = 12) and 1039.05±88.99 pN for 60 sec (n = 12). The differences were significant when compared with SCF (152.00 ± 19.43 pN for 0.5 sec, p< 0.05, n = 12; 457.87 ± 96.15 pN for 10 sec, p< 0.01, n = 12; and 629.57 ± 57.04 pN for 60 sec, p< 0.01, n = 12) or CF (154.25 ± 9.98 pN for 0.5 sec, p< 0.05, n = 12; 437.84 v 74.71 pN for 10 sec, p< 0.05, n = 12; and 676.12 ± 95.59 pN, p< 0.05, n = 12) subgroups, respectively. Although the lowest adhesion forces were detected in SCF and CF subgroups, there was no statistically significant difference between these two groups for all three time points. Adhesion forces between MB231 cells and Mgrt HPMEC (237.49 ± 42.36 pN for 0.5 sec, n = 12; 658.65 ± 126.02 pN for 10 sec, n = 12; and 1010.09 ± 149.22 pN for 60 sec, n = 12) for all contact times were almost equal to those in NCF subgroup.

### Individual ligand-receptor ruptures analysis

**Fig 8:**
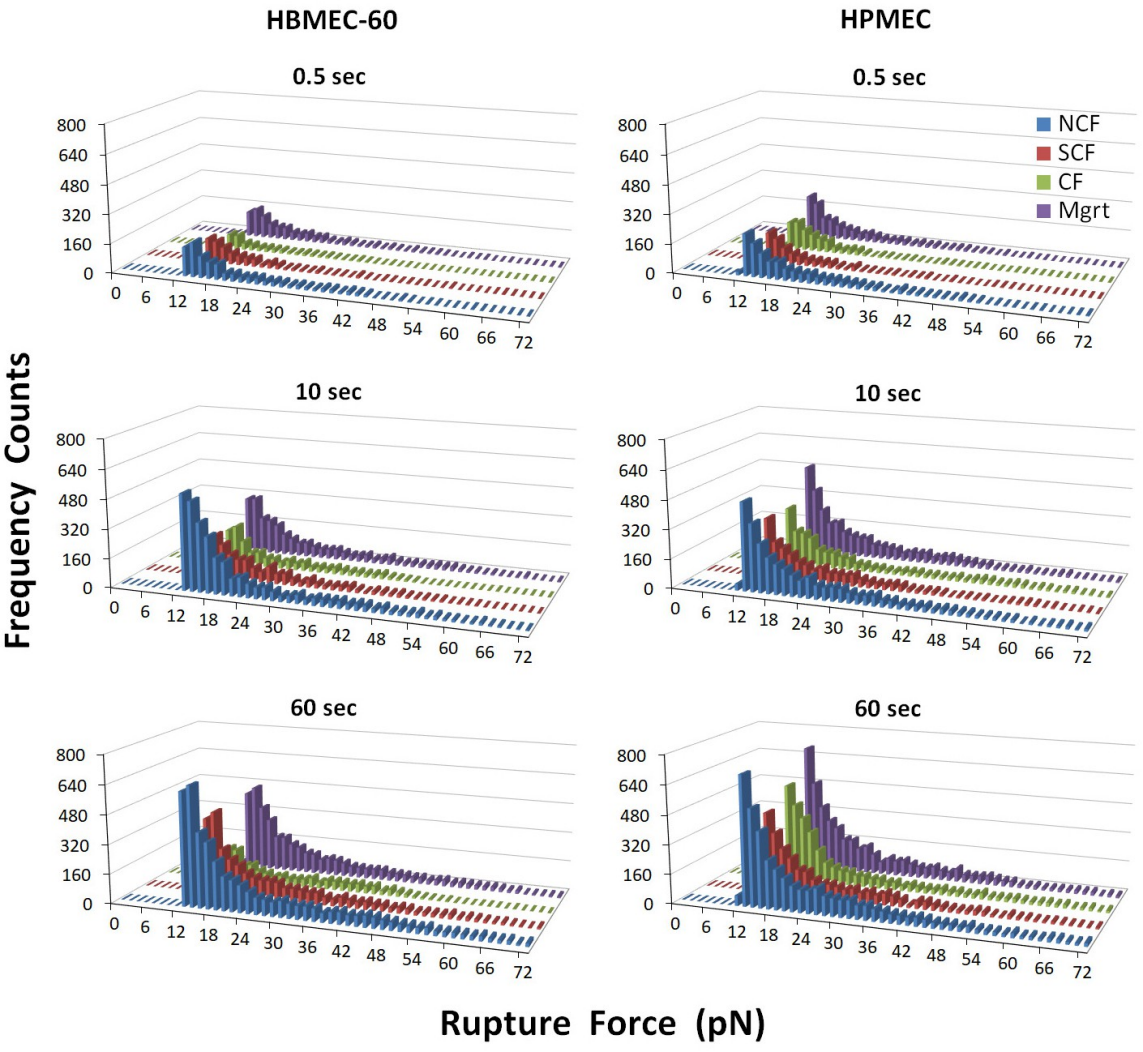
Distributions of rupture forces from MB231/HBME-60 and MB231/HPMEC adhesion measurements in four EC growing states. For all three contact periods, the number of rupture forces were the greatest in NCF HBMEC-60 cells (1097, 3293 and 4669 counts for 0.5, 10 and 60 sec, respectively), reduced in the SCF cells, and lowest (2.59, 2.58 and 3.35 times less than those in NCF cells) in CF HBMEC-60 cells. Counts of rupture forces in Mgrt HBMEC-60 were slightly lower than those in the NCF cells, but higher than in the SCF cells. In HPMEC, the NCF cells for three contact times demonstrated the largest frequency counts (1423, 3108 and 4519 for 0.5, 10 and 60 sec, respectively) in four growing states. Mgrt cells showed very close counts to NCF cells. In each panel, Frequency Counts are plotted on Y axis and Rupture Forces (pN) on X axis.

**Fig 9:**
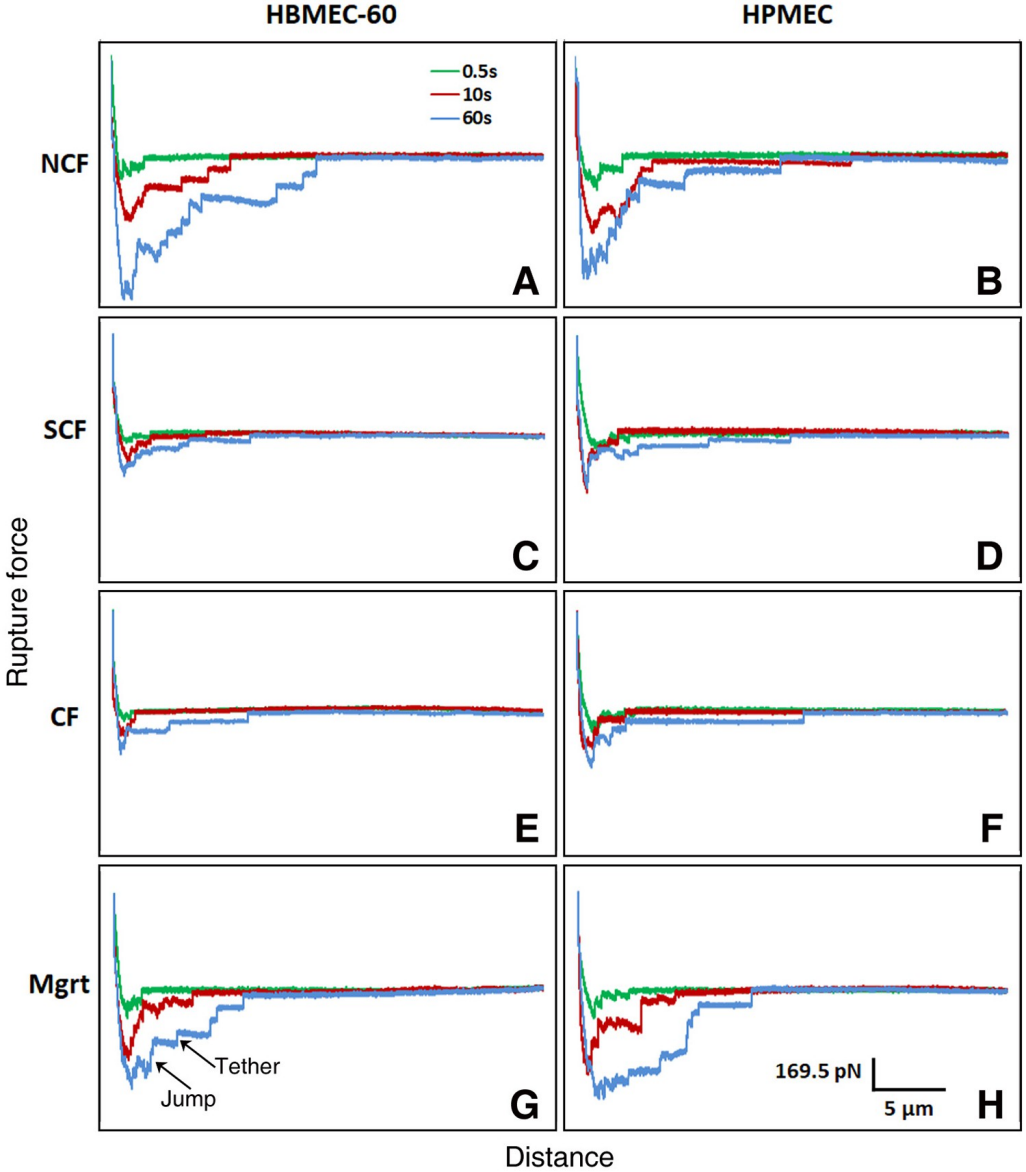
Typical AFM detachment curves of MB231 cells interacting with HBMEC-60 (A, C, E and G) and HPMEC (B, D, F and H) cells at NCF (A and B), SCF (C and D), CF (E and F) and Mgrt (G and H) states for 0.5 (green), 10 (red) and 60 (blue) sec cell contact times. For both HBMEC-60 and HPMEC note significant increase in overall adhesion force between MB231 and ECs in NCF and Mgrt states compared to SCF and CF cells most noticeable for 10 and 60 sec cell contact times and accompanied by obvious increase in the number of both jumps and tethers (indicated with arrows in G) suggesting the involvement of both, receptors that are tightly associated (jumps) and weakly or not associated (tethers) with the cytoskeleton. Scale for Y and X axes are shown in H.

To gain further insights into the dynamic changes of cancer cell-EC adhesive interactions, we have analyzed rupture events that represent breaking of individual ligand-receptor interactions (adhesion bonds) between cancer cells and ECs, contributing to the total adhesion forces between the two cell types. Fig 8 shows distribution of rupture forces from measurements of MB231/HBME-60 and MB231/HPMEC adhesion in four EC growing states. In the MB231/HBME-60 group for all three cell-cell contact periods, the rupture counts were greatest in NCF cells (1097, 3293 and 4669 counts for 0.5, 10 and 60 sec, respectively), reduced in the SCF, and lowest in the CF cells (correspondingly 2.59, 2.58 and 3.35 times less than those in NCF). The numbers of rupture forces in Mgrt HBMEC-60 were 0.99 (0.5 sec), 0.72 (10 sec) and 0.79 (60 sec) times less than those for the NCF HBMEC-60 cells, but greater in value than in the SCF group. In MB231/HPMEC group, the NCF cells also showed the largest frequency counts for all three contact periods (1423, 3108 and 4519 for 0.5, 10 and 60 sec, respectively). The numbers of ruptures in SCF and CF subgroups were similar at the respective time points, and both were 1.4–1.7 times lower than the number of ruptures in NCF. The Mgrt subgroup (1321, 3068 and 4389, respectively) had an equivalent number of ruptures as the NCF cells at the same cell contact times. The distribution patterns of rupture forces at all time points in both EC groups were similar and no distinct peak shifts were observed (see S2 File for a detailed breakdown of ligand-receptor rupture forces for each force bin as well as for 2D graphs of rupture force distribution for each experimental setup). However, in addition to changes in the number of ruptures, the stronger rupture forces decreased in CF ECs, as compared with the NCF. These data are consistent with the changes in total MB231/EC adhesion forces at different EC confluency, and suggest that the changes in both the number and the strength of the individual ligand-receptor interactions contribute to changes in total adhesion forces between tumor and endothelial cells. A further look at the typical detachment curves from our adhesion experiments (Fig 9) reveals the presence of both jumps i.e., rupture events immediately associated with force increase and presumably corresponding to receptors tightly anchored to the cytoskeleton [13, 32], and tethers i.e., rupture events preceded by a plateau with no visible change in force before the adhesin bond brake and associated with the molecules that are not or weakly associated with actin cell cortex [13, 32]. It is evident that in experiments with NCF (Fig 9A and 9B) and Mgrt (Fig 9G and 9H) cells the number of both jumps and tethers is higher compared with SCF (Fig 9C and 9D) and CF (Fig 9E and 9F) cells. This suggests that increases in adhesive interactions with receptors tightly anchored to the cytoskeleton as well as with the ones rather weakly or not associated with actin cortex are both contributing to the overall strengthening of adhesion with tumor cells in NCF and Mgrt cells (see Discussion).

### MB231-EC adhesions inversely correlates with EC stiffness

Our results show that for both endothelial cell lines the strength of their adhesive interactions with MB231 tumor cells vary significantly dependent on their growth state (Fig 6) so that both HBMEC-60 and HPMEC exhibit stronger adhesion with MB231 when they are in a single cell state (NCF and Mgrt) compared with the growth states when ECs interact with each other (SCF and CF). Remarkably, EC elasticity also depends on their growth state (Fig 4). That is, for both HBMEC-60 and HPMEC their stiffness is greater when cells are in SCF and CF states compared with the single cell states (NCF and Mgrt). Thus, our next question was whether dynamic changes in EC elasticity and the strength of their adhesive interactions with tumor cells are correlated.

**Fig 10:**
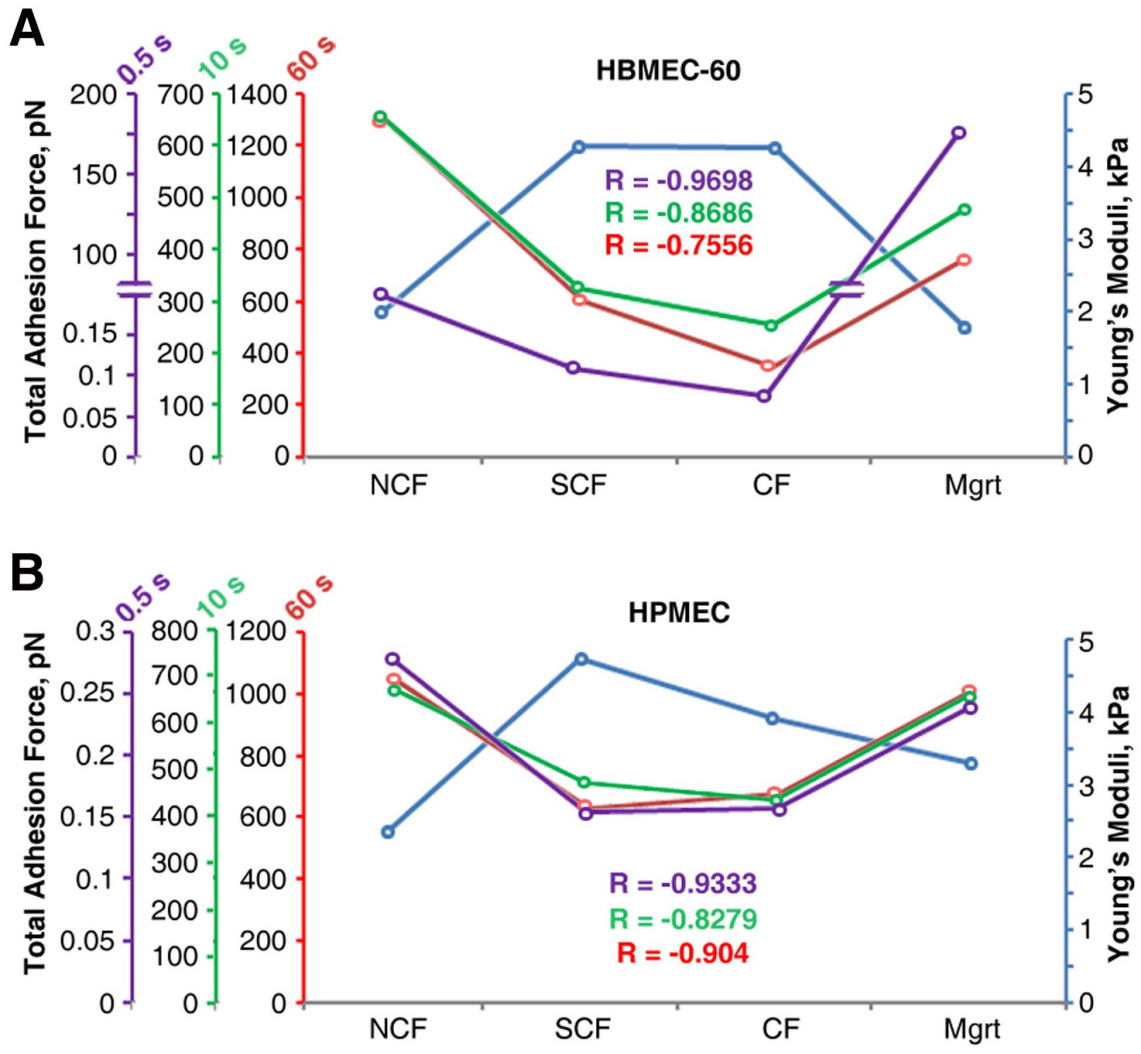
Regression analysis of the relationship between EC elasticity and total strength of adhesion with MB231 cells at NCF, SCF, CF and Mgrt states for three (0.5, 10 and 60 sec) cell contact times. For both HBMEC-60 (A) and HPMEC (B) there is a very strong negative correlation between elasticity and total strength of adhesion with MB231 at all three cell contact times with correlation coefficient (R) ranging from -0.76 to -0.97 for HBMEC-60 and from -0.83 to -0.93 for HPMEC.

The regression analysis demonstrated that for HBMEC-60 there was a very strong negative correlation between the EC elasticity and the strength of their adhesion with tumor cells at all three contact times tested (Fig 10A). The correlation coefficient R was -0.97, -0.87 and -0.76 for 0.5s, 10s, and 60s contact times respectively.

Similarly, a very strong reciprocal correlation between EC elasticity and the strength of EC adhesion with tumor cells was evident for HPMEC (Fig 10B). The correlation coefficients were -0.93 for 0.5s contact time, -0.83 for 10s contact time, and -0.9 for 60s contact time.

## Discussion

In this study, using atomic force single cell spectroscopy, we collected novel information regarding changes in elasticity of ECs as a function of cell confluence and dynamics of their adhesive interactions with tumor cells. We investigated mechanical properties (stiffness) in two types of ECs at different degrees of confluence. Specifically, we have detected Young’s moduli of 2.03 kPa in human bone marrow HBMEC-60 cells and 2.33 kPa in human pulmonary microvascular ECs HPMEC in NCF state, both of which are consistent with mean ranges of 2.0–6.67 kPa in ECs reported previously by others [24, 27, 29]. Since multiple factors such as cell type, environment, aging and disease state affect cell mechanical properties [30, 31, 38, 39], we also addressed the question of whether or not the state of confluence (growth status) is a factor influencing the cellular elastic properties. We found that EC stiffness significantly increased with confluence and the establishment of cell-cell contacts, but decreased as they transitioned to a single cell state in scratch assay (Fig 4). Cell stiffness is mainly determined by the state of the actin cytoskeleton, it is therefore dependent on activation state, ratio of actin polymerization/depolymerization, and stress fibers spatial organization and distribution [24, 27, 40]. Topographic and fluorescent imaging of cortical cytoskeleton networks revealed that the stress fibers in SCF and CF ECs were denser and thicker than in the NCF state (Figs 5 and 6). This suggests that increased density and size of actin stress fibers is likely related to increased polymerization and intracellular remodeling of the actin cytoskeleton (i.e., F-actin formation by polymerization of G-actin) [41].

Comparisons of cellular mechanical properties as a function of growth and confluence have been investigated previously in very few studies. One study performed on cerebral endothelial cells by Végh et al. [25], demonstrated that the Young’s moduli in cerebral endothelial cells were 4 ± 1 kPa at sub-confluence and 3 ± 1 kPa at confluence, and there was no difference between the two growth states. This result is in agreement with our observations showing no difference in stiffness between SCF and CF ECs. However, the author did not report any results on the NCF EC state. Another investigation using a different cell type, Vero cells (African green monkey Cercopitecus aethiops kidney cell line), revealed that the Young’s moduli of confluent cells were 1.4 to 1.7 times lower than those of single non-confluent or scratch migrating cells [42]. Thus, in addition to confluency, cell type should be also a consideration for future studies in which cell stiffness is an important variable.

Next, we characterized adhesive interactions of MB231 with two types of ECs in different EC growth states. We evaluated HBMEC-60 and HPMEC cells because they were sourced from human bone marrow and lungs, two organs which are common sites for breast cancer metastasis. Our results demonstrated that the strength of adhesion between cancer and ECs decreased as ECs developed cell-cell contacts and became more confluent, but increased when ECs switched to single state in scratch assay. The increased stiffness and decreased adhesion in confluent ECs are most likely coupled. Indeed, the regression analysis (Fig 10) demonstrated that for both endothelial cell lines, the total strength of adhesion exhibits a very strong negative correlation with elasticity at all three cell contact times tested. This does not establish, however, a causative relationship between EC stiffness and adhesion, but implies that changes in EC stiffness associated with their cytoskeleton reorganization may also lead to, or coincide with changes in the repertoire of cell adhesion molecules expressed on EC outer membranes and available for interactions with tumor cells. Thus, a number of important questions will need to be addressed in our future studies: 1). How ECs cell surface adhesion molecule repertoire changes, when they transition from NCF to SCF to CF and Mgrt states? 2) Are changes in EC cell surface adhesion molecule expression a direct consequence of actin cytoskeleton remodeling, or do they simply coincide with the latter while ECs change their growth and confluence state? At this time, we speculate that immature actin stress fibers in NCF and Mgrt ECs may be associated with an increased frequency/expression/abundance of adhesion molecules available for cell-cell interactions compared to SCF and CF EC states characterized by more abundant and organized stress fibers (Figs 5 and 6). In addition, the possibility exists that even though the SCF and CF ECs have many more actin stress fibers, the associated adhesion molecules could be largely occupied by interactions with neighboring ECs and consequently less available for adhesive interactions with tumor cells. Also, as detachment curves from NCF and Mgrt cell experiments exhibit significantly more of both jumps and tethers compared with SCF and CF cells (Fig 9), it implies that increases in adhesive interactions with both, receptors that are tightly associated (jumps) and weakly or not associated (tethers) with the cytoskeleton, are involved in the overall increase in the strength of adhesion between NCF and Mgrt ECs with tumor cells.

Classically, actin stress fibers are intimately associated with focal adhesions, macromolecular assemblies transmitting mechanical forces and regulatory signals between the cell and extracellular matrix (ECM). Our previous [4, 18] and more recent works unambiguously identify tumor-associated Thomsen-Friedenreich antigen and endothelium expressed Galectin-3 and α3β1 integrin involvement in cancer cell-EC adhesion [22, 23] and induction of focal adhesion type macromolecular signaling complex formation downstream of these interactions [22]. In addition to α3 and β1, several other integrins could be actively involved in tumor cell-EC adhesion [22], suggesting the potential importance of EC stress fibers and associated adhesion molecules in metastasis-associated cancer cell-EC adhesive interactions. In addition to integrins, dynamic changes in other endothelium expressed adhesion molecules could be involved as well [43]. For example, Haddad et al. using human umbilical vein endothelial cells (HUVECs) demonstrated that after tumor cell coculturing with HUVECs for 5 hours, an increase in endothelial ICAM-1 (Inter Cellular Adhesion Molecule-1), VCAM-1 (Vascular Adhesion Molecule-1) and E-selectin is observed [44]. However, considering a much shorter timeframe (up to 60 sec) of tumor cell interactions with ECs in our study, it is unlikely that this scenario took place in our experiments.

The detailed analysis of rupture events representing breaking of individual ligand-receptor interactions between ECs and tumor cells contributing to the total adhesion forces between the two cell types (Fig 8) demonstrated a significant increase in both the total number of rupture events and the number of stronger rupture forces in single (NCF and Mgrt) ECs compared to SCF and CF cells. These results, coupled together with our previous observations [20], support further our current hypothesis and provides a framework for future investigations aimed at identifying the underpinning molecular mechanisms driving blood borne metastatic tumor cell adhesive interactions with vascular endothelium.

In summary, we have used AFM single cell force spectroscopy to characterize elastic properties of bone marrow endothelial cells and pulmonary microvascular endothelial cells in different growth states and degrees of confluence as well as their adhesive interactions with human breast carcinoma cells at three cell contact times. Confluent, subconfluent and single/migrating EC states were used as *in vitro* models representing the different states of the blood vessel endothelial lining (intact, damaged and recovering through wound healing). Our work suggests that the integrity of the endothelial barrier is an important factor in reducing metastatic tumor cell ability to lodge in distant organ vasculature and develop clinically relevant secondary tumors. For example, human organs with sinusoidal capillaries with discontinuous EC lining such as bone marrow and liver are frequently targeted by cancer metastasis However, as this study was performed entirely *in vitro*, it is important to acknowledge that the model employed in these experiments is only an approximation of the *in vivo* metastasis-associated situations. Whether and to what degree the findings presented in this study are relevant to in vivo process of hematogenous cancer metastasis remains to be elucidated.

## Supporting information

S1 File Comparisons of HBMEC-60 and HPMEC elasticities at non- (NCF), sub- (SCF), full- (CF) confluent and migrating (Mgrt) growing status. (XLSX)

S2 File Adhesion of MB231 to HBMEC-60 and HPMEC cells at non- (NCF), sub- (SCF), full-confluent (CF), and migrating (Mgrt) growing states. (XLSX)
